# Microscopic Colitis: An Underestimated Disease of Growing Importance

**DOI:** 10.3390/jcm13195683

**Published:** 2024-09-24

**Authors:** Kamil Rutkowski, Karina Udrycka, Barbara Włodarczyk, Ewa Małecka-Wojciesko

**Affiliations:** Department of Digestive Tract Disease, Medical University of Lodz, 90-647 Lodz, Poland; karina.udycka@stud.umed.lodz.pl (K.U.); barbara.wlodarczyk@umed.lodz.pl (B.W.); ewa.malecka-panas@umed.lodz.pl (E.M.-W.)

**Keywords:** inflammatory bowel disease, lymphocytic colitis, collagenous colitis, chronic diarrhoea, microscopic colitis

## Abstract

The aim of this paper is to raise awareness of MC as a clinically significant condition and to highlight its under-recognition, risk factors, diagnosis, management, and complications. This paper underlines the diagnostic and therapeutic challenges associated with the often nonspecific symptoms of MC. In order to create this article, we reviewed available articles found in the PubMed database and searched for articles using the Google Scholar platform. Microscopic colitis (MC) is a chronic inflammatory bowel disease, classified into three types: lymphocytic, collagenous, and unspecified. The average age of onset of MC is around 62–65 years and the disease is more common in women than men (nine times more common). The main symptom of MC is watery diarrhoea without blood, other symptoms include defecatory urgency, faecal incontinence, abdominal pain, nocturnal bowel movements, and weight loss. Once considered a rare disease, MC is now being diagnosed with increasing frequency, but diagnosis remains difficult. To date, a number of causative factors for MC have been identified, including smoking, alcohol consumption, medications (including NSAIDs, PPIs, SSRIs, and ICPIs), genetic factors, autoimmune diseases, bile acid malabsorption, obesity, appendicitis, and intestinal dysbiosis. It may be difficult to recognize and should be differentiated from inflammatory bowel diseases (Crohn’s disease and ulcerative colitis), irritable bowel syndrome (IBS), coeliac disease, infectious bowel disease, and others. Diagnosis involves biopsy at colonoscopy and histopathological evaluation of the samples. Treatment consists of budesonide oral (the gold standard) or enema. Alternatives include bile acid sequestrants (cholestyramine, colesevelam, and colestipol), biologics (infliximab, adalimumab, and vedolizumab), thiopurines, methotrexate, and rarely, surgery.

## 1. Introduction

Microscopic colitis (MC) is an inflammatory disease of the bowel that is a common cause of chronic, watery diarrhoea without blood (up to 10 times per day, one-third of which occurs at night) [1]. This results in considerable discomfort for those affected and a reduction in their health-related quality of life (HRQoL) [1]. In the majority of cases, no lesions are observed during endoscopy. Only after performing a biopsy and conducting a histopathological examination can the microscopic changes characteristic of MC be detected. It is therefore imperative to obtain biopsy specimens from patients presenting with chronic diarrhoea, even in the absence of macroscopic changes. From a histological perspective, two principal categories of MC can be identified: lymphocytic and collagenous colitis. Lymphocytic colitis (LC) is associated with an increased number of intraepithelial lymphocytes (more than 20 per 100 epithelial cells, with a normal range of 3–5 lymphocytes), while collagenous colitis (CC) is associated with a thickened collagen layer at the base of the epithelial cells (10–100 µm, with a normal range of 5–7 µm) [2,3,4]. Moreover, some authors propose a third form, incomplete microscopic colitis (MCi), characterized by a collagen thickness of less than 10 µm and a lymphocyte count of less than 20. This distinction proved necessary since although there are non-normal findings in the histopathology, the criteria for LC and/or CC are not fully met [5,6]. The pathologies are considered subtypes of one disease due to their similar clinical manifestations, aetiology, pathogenesis, and therapeutic approach. The UEG and EMCG (United European Gastroenterology and European Microscopic Colitis Group) guidelines advise that multiple biopsy specimens should be collected from both the left and right colon. Although macroscopic alterations are not a defining feature of the disease, there have been reports of linear ulcerations, mucosal ruptures, erythema, and colonic oedema [7].

The aetiology of the disease remains unclear. The aetiological factors include autoimmune diseases (such as ankylosing spondylitis (AS), psoriasis, Hashimoto’s disease, Graves–Basedow disease, and type 1 diabetes), as well as medications used (such as NSAIDs (Non-Steroidal Anti-Inflammatory Drugs), PPIs (Proton Pump Inhibitors), SSRIs (Selective Serotonin Reuptake Inhibitors), and ICPIs (immune check-point inhibitors)). Additionally, HLA (Human Leukocyte Antigen) variations, genetic factors (metalloproteinase gene polymorphism and 5-HTTLPR gene polymorphism), as well as intestinal dysbiosis, bile acid malabsorption, and obesity have been identified as potential etiological factors. Additionally, it has been found that the risk of MC is increased following appendectomy [8,9,10].

At present, a national registry of patients diagnosed with MC is maintained in Poland. The registry was established by the Microscopic Colitis Section of the Polish Society of Gastroenterology (PTG-E). The Section is chaired by Professor Wojciech Marlicz. The registry is currently being coordinated at eight Reference Centres throughout the country with the objective of accurately analysing the clinical data of patients who will be included in the 10-year surveillance programme. The registry comprises a questionnaire completed by the physician based on an interview with the patient. This questionnaire covers the diagnosis of MC, its subtype, the most common symptoms, concomitant diseases, and the implemented treatment. On an annual basis, the clinical data will be complemented with possible new events, such as the occurrence of MC exacerbations and the pharmacotherapy changes. The establishment of a national database aims to facilitate further research into MC, enhance earlier and more precise diagnosis, widen the awareness of the disease in medical staff and society, create national unification of disease management, and ensure implementation of the current and most efficacious treatment. Current data from the MC registry have not yet been published, but we believe that it will help to improve the recognition and uniform management of this pathology within the country. In connection with the national MC registry, conferences are planned to promote knowledge and reports on the number of registered and the dynamics of the increase in the frequency of occurrence, which will be assessed and interpreted. The Polish Microscopic Colitis Registry is used to transmit, collect, and process medical data from across the country in the context of patients diagnosed with MC monitored within the National Medical Register.

The aim of this paper is to raise awareness of MC as a clinically significant condition and to highlight its under-recognition, risk factors, clinical presentation, diagnosis, management, and complications. This paper underlines the diagnostic and therapeutic challenges associated with the often nonspecific symptoms of MC. In order to create this article, we reviewed available articles found in the PubMed database and searched for articles using the Google Scholar platform.

MC remains a condition often overlooked in the diagnosis of diseases presenting with chronic diarrhoea. This is due to the limited awareness of MC among medical staff, with this disease often being confused with more common IBS and other pathologies with chronic diarrhoea. This condition is also frequently overshadowed by other inflammatory bowel diseases.

MC symptoms overlap with those of IBS and up to 50% of patients with this disease meet the Rome III criteria for IBS [11,12]. A recent meta-analysis of MC in IBS patients shows an MC prevalence of 7.1–7.4% [13]. On the other hand, the prevalence of microscopic colitis in patients with diarrhoea from other causes was as high as 10.9%. In addition, taking biopsies from normal-appearing colonic mucosa is often omitted by endoscopists.

Although knowledge about this disease is rapidly advancing, there are still difficulties in its proper diagnosis and treatment. Challenges in the early diagnosis of MC include nonspecific clinical symptoms and a normal endoscopic appearance of the colon [14]. Neither laboratory abnormalities nor biomarkers, such as calprotectin, provide significant diagnostic value [15]. The selection of biopsy locations and the number of samples taken are crucial for accurate diagnosis. Cellularity and collagen thickening can vary across different areas of the colon, meaning that biopsies from unaffected regions may lead to diagnostic errors [16]. It is also important to note that the clinical presentation can change over time during the natural course of the disease, which may be considered a distinctive feature of MC. Additionally, diagnostic errors may be due to various biopsy sites, different staining techniques, and interpretation by pathologists of different experience [14].

In this paper, we have attempted to compile and systematize the current knowledge regarding MC, which is crucial for improving the effectiveness of MC diagnosis, and treatment and reducing the duration of symptoms. In order to prepare this article, we reviewed available articles found in the PubMed database and searched for articles using the Google Scholar platform.

## 2. Epidemiology

The estimated mean incidence rate of both MC subtypes in Europe is approximately 11.4 cases per 100,000 person-years [7]. The estimated average incidence of CC is 4.9 cases per 100,000 individuals per year, while the estimated average incidence of LC is five cases per 100,000 inhabitants per year [7]. The highest incidence rates were reported in Denmark, reaching 24.3 cases per 100,000 person-years [17]. The incidence of MC is reported to be comparable to or even higher than that of inflammatory bowel disease (IBD). The increased incidence of MC is most likely associated with the excessive use of medications that may contribute to the induction of this condition, improvements in endoscopic diagnostic techniques, and the gradually growing awareness of both patients and healthcare providers regarding this disease entity [17].

The issue of differences in the incidence of MC across various ethnic groups is controversial. Some studies suggest that MC is more prevalent among individuals of the Caucasian population compared to the Asian population (in Japan, the JADER study [18] reported only 161 cases of MC between 2004 and 2021). Very few cases have been reported among individuals of African descent. In 2021, Oluyemi et al. [19] reported only three cases. Frequent misdiagnoses of MC further complicate obtaining precise epidemiological data. Additionally, the low clinical awareness of MC and limited interest in the industry results in a low number of epidemiological studies [2,7,14,17].

The prevalence of MC in women is higher than in men and is approximately 3 to 2, meaning that for every three women with MC, there are two men with the same disease. Furthermore, some studies show that the prevalence of CC can be up to nine times higher in women than in men, depending on the size of the study group [2,14]. The mean age of onset of CC is 64.9 years, while LC is 62.2 years [20]. It is noteworthy that in only 25% of LC cases, the diagnosis is made before the patient reaches the age of 45 [2]. There have also been single cases of MC among children noted in the literature [21,22].

Although the impact of diet on IBD clinical course and the role of diet–microbial–immune system interactions in the development of the disease has been the subject of numerous studies [23,24], little is known about the role of diet in MC. Nevertheless, the influence of calcium intake, alcohol consumption, and smoking on MC development has been reported, as discussed in Section 3 [25,26]. Nonetheless, the limited number of studies highlights the need for further research in this area.

## 3. Risk Factors

### 3.1. Smoking and Alcohol

It has been demonstrated that both past and current smoking are associated with an increased risk of developing lymphocytic colitis (LC) and collagenous colitis (CC) [25,27,28]. Moreover, a study by Burke et al. [27] indicated that the risk of MC increases with an increasing number of pack-years and that this risk decreases five years after smoking cessation. It has been hypothesised that the disease may manifest up to a decade earlier among smokers than in non-smokers [28].

It is suggested that the development of MC in response to smoking is facilitated by modifications of the intestinal flora. An increase in the number of anaerobic bacteria may result in the stimulation of transforming growth factor beta (TGF-β) production, which in turn leads to an increase in collagen deposition in the colon wall. Moreover, dysbiosis affects the regulation of immune system function and the modification of the epithelial barrier. The immune system is dysregulated, resulting in the excessive activation of CD8+ T lymphocytes, which in turn causes damage to the intestinal epithelium. The integrity of the epithelial barrier is compromised, resulting in increased permeability to antigens and bacteria and enhanced inflammation [27,29].

The consumption of substantial quantities of alcohol with a high alcohol content has been identified as a risk factor for MC. It has been demonstrated that ethanol and its metabolite, acetaldehyde, cause an increase in the permeability of the epithelial barrier of both the small and large intestine, which in turn promotes inflammation. Other potential mechanisms include the induction of oxidative stress and the activation of pro-inflammatory cell signalling pathways [25].

### 3.2. Intestinal Dysbiosis

Disturbances in the composition of the intestinal microflora have been identified as a contributing factor in the development of MC. The available evidence indicates that the risk of CC increases threefold and persists for a minimum of three years following a *Clostridioides difficile* infection [30]. Microorganisms that have been identified as playing a specific role in the development of CC include *Clostridioides difficile*, noroviruses, and to a lesser extent, *Escherichia* spp., *Campylobacter concisus*, and *Campylobacter jejuni* [10,30]. This is likely attributable to the production of pro-inflammatory cytokines by the microorganisms and a reduction in the number of protective bacteria. Protective bacteria include *Lactobacillus* spp., *Bifidobacterium* spp., and *Akkermansia muciniphila*, which contribute to intestinal protection through the production of lactic acid, bacteriocins, and short-chain fatty acids, supporting the integrity of the intestinal barrier [27,31,32].

Furthermore, few cases of MC development in patients following faecal microbiota transplantation (FMT) have been documented. It is hypothesised that following faecal transplantation into an organism with an abnormal immune response, inflammation-inducing lymphocytes migrate into the gut. In addition, the sudden increase in bacterial diversity is postulated to increase the amount of bacterial metabolites, which may also contribute to the development of MC [33].

A study by Verhaegh et al. [34] demonstrated that the concurrent administration of PPIs and NSAIDs markedly elevated the likelihood of developing MC compared to individuals who were prescribed only one or none of these medications. The elevated risk is attributed to the combined effects of those drugs. Proton Pump Inhibitors (PPIs) alter the pH of the gastrointestinal tract, resulting in alterations in the gut microbiome. On the other hand, NSAIDs reduce the synthesis of prostaglandins, which protect the gastrointestinal mucosa from damage. Additionally, they stimulate the production of pro-inflammatory cytokines (TNFα, IL-1β, IL-6, and INFγ) and increase intestinal permeability, and thus induce the translocation of toxins, antigens, and bacteria. In MC, the translocation of toxins, antigens, and bacteria across the intestinal epithelial barrier can lead to inflammation and immune dysregulation. The barrier becomes compromised, allowing these substances to penetrate into deeper layers of the colon, thus triggering immune responses. This translocation can result in the activation of immune cells, including macrophages and T cells, which release inflammatory cytokines. Such inflammation can perpetuate damage to the mucosal barrier, leading to a cycle of inflammation, chronic diarrhoea, and further progression of MC [35,36].

### 3.3. Non-Steroidal Anti-Inflammatory Drugs (NSAIDs)

The precise mechanism by which NSAIDs, including ASA (aspirin, acetylsalicylic acid), contribute to MC remains unclear. One of the hypothesized mechanisms is described in Section 3.2 “Intestinal Dysbiosis”.

The findings of Bjurström et al. [35] suggest that NSAID use in individuals with MC may potentially contribute to relapse and the necessity for an additional cycle of budesonide treatment. In contrast, patients who used ASAs, SSRIs (Selective Serotonin Reuptake Inhibitors), PPIs (Proton Pump Inhibitors), and statins did not demonstrate an elevated risk of relapse.

### 3.4. Proton Pump Inhibitors (PPIs)

It is hypothesized that PPIs may precipitate the onset of the disease due to an increase in gastric pH and subsequent intestinal dysbiosis and electrolyte imbalance. Conversely, some authors posit that PPIs may directly induce intraepithelial lymphocytosis [37,38]. It has been observed that lansoprazole use causes an elevated risk of developing MC [38].

Two meta-analyses were conducted in large cohorts of patients, with the results demonstrating a moderate increase in the risk of developing MC as a consequence of PPI use. The studies included by Tarar et al. did not analyse the dose, type, or subtypes of PPI, nor the types of MC. When using a control group comprising individuals with chronic diarrhoea other than MC, the risk of developing MC was even reduced. This indicates that this problem is complex and requires further research [37,39].

### 3.5. Selective Serotonin Reuptake Inhibitors (SSRIs)

It is assumed that an increase in serotonin release, resulting from the stimulation of 5-HT3 receptors in enterochromatophilic cells, may be a potential cause of watery diarrhoea [37,40]. A robust correlation has been established between SSRI administration and the emergence of LC. The elevated risk of LC in SSRI users is presumably associated with the potential for these drugs to enhance intestinal barrier permeability. Studies suggest that clinicians should be aware of the potential risk of LC in patients taking SSRIs and consider alternative therapeutic options for patients presenting with diarrhoea [41,42]. Furthermore, there have been reports of CC induction by escitalopram [40,43].

### 3.6. Immune Check-Point Inhibitors (ICPIs)

ICPIs are a class of drugs that act as inhibitors of proteins such as programmed cell death protein 1 (PD-1), programmed death ligand 1 (PD-L1), and cytotoxic T cell antigen 4 (CTLA-4). They are employed with increasing frequency in the treatment of a range of cancers, including melanoma, non-small-cell lung cancer (NSCLC), kidney cancer, bladder cancer, Hodgkin’s lymphoma, colorectal cancer, gastric cancer, hepatocellular carcinoma, oesophageal cancer, and breast cancer [44].

The drugs belonging to the ICPI class that have the potential to cause MC are those that inhibit CTLA-4, PD-1, and PD-L1 [45,46]. It is hypothesised that the inhibition of PD-1, PD-L1, and CTLA-4 may result in the activation of Th1 and Th17 lymphocytes, leading to an increased production of pro-inflammatory cytokines. Furthermore, the influence of ICPIs has been observed to result in the enhanced activation of B lymphocytes, which in turn leads to an increased production of antibodies involved in inflammatory and autoimmune processes. Additionally, ICPIs have been observed to induce heightened activity in Gram-positive bacteria, which is linked to alterations in the microbiome in response to ICPIs. Such bacteria have the potential to enhance the immune response, which may result in the development of inflammation within the gut [47].

The incidence of MC with the use of CTLA-4 inhibitors is 3.4–22%. With the combination of CTLA-4 inhibitors and PD-1, the incidence of MC is 0.7–12.8%, and 0.7–2.6% with PD-1 or PD-L1 inhibitors. Furthermore, the risk of MC is additionally increased in individuals of Caucasian ethnicity and those with an existing diagnosis of IBD [45].

### 3.7. Other Medications

A number of other drugs have been linked to the possibility of inducing MC. These include angiotensin-converting enzyme inhibitors (ACEIs), β-blockers, statins, bisphosphonates, angiotensin II receptor blockers (ARBs) and topiramate. However, the effect of these drugs is uncertain [37,38,48,49,50].

### 3.8. Genetic Factors

The HLA-DQ2 haplotype, as observed in coeliac disease, has been linked to an elevated risk of MC and enteropathy. HLA-DQ2 is a factor that is expressed on the surface of antigen-presenting cells to T lymphocytes, resulting in their activation and the subsequent production of anti-inflammatory cytokines [51]. Matrix metalloproteinase (MMP) gene polymorphisms have been linked to an elevated risk of CC, potentially due to aberrant collagen degradation [9].

Furthermore, the polymorphism of the gene encoding interleukin-6 has been identified as a potential risk factor for MC. The IL-6-174 GG genotype has been demonstrated to be associated with an elevated risk of MC, which leads to augmented IL-6 production [51,52].

The serotonin-transporter-linked polymorphic region (5-HTTLPR) gene (LL genotype of the 5-HTTLPR gene), which is part of the promoter of the gene encoding the serotonin transporter, may contribute to the development of MC. This polymorphism leads to increased 5-HT secretion, which may contribute to diarrhoea and abdominal pain. Sikander et al. [53] investigated the correlation between 5-HTTLPR polymorphism and the increased prevalence of MC through a genotypic analysis of patients diagnosed with MC compared to healthy subjects.

### 3.9. Autoimmune Disease

Microscopic colitis (MC) is strongly associated with the development of autoimmune diseases, affecting nearly 38.8% of patients with MC compared to 19.1% of patients with inflammatory bowel disease (IBD). The most commonly co-occurring autoimmune conditions include coeliac disease, Hashimoto’s thyroiditis, rheumatoid arthritis (RA), Sjögren’s syndrome, psoriasis, and type 1 diabetes mellitus [20,54,55].

Wildt et al. demonstrated a significant correlation between the presence of coeliac disease and an elevated risk of developing MC, with an odds ratio of 10 times higher compared to individuals without the condition [54]. The frequent coexistence of coeliac disease and MC (particularly of the LC type) is linked to shared pathogenetic mechanisms. Both conditions have an autoimmune (HLA-DQ2) and inflammatory basis and are associated with immune system dysregulation. It is crucial that patients with coeliac disease presenting with chronic diarrhoea undergo a colonic biopsy to diagnose MC, as this is key for implementing an effective treatment plan [56].

The underlying mechanism of autoimmune disease coexistence in MC is likely related to chronic inflammation confined to the colon. This persistent inflammation may lead to immune system dysregulation. Unlike IBD, MC rarely causes extraintestinal symptoms but is more frequently associated with autoimmune disorders. Studies have shown that the inflammation in MC is mediated by immune cells, particularly T lymphocytes, which may trigger autoimmune responses. Additionally, gut dysbiosis, which plays a role in the pathogenesis of MC, may contribute to the development of autoimmune responses through the induction of inflammation and activation of T lymphocytes (see Section 3.2 “Intestinal Dysbiosis”) [55].

### 3.10. Disorders of Bile Acid Absorption

In a study by Fernandez et al. [57], it was found that the disorders of bile acid absorption have been associated more often with LCs than CCs. In a study by Lan et al. [58], the presence of a marker of bile acid malabsorption was observed in both types of MC. An association was demonstrated between MC and aberrant results on the Selenium Homocholic Acid Taurine (SeHCAT) test, which is employed to evaluate anomalies in bile acid absorption (low bile acid absorption). It seems reasonable to posit that bile acids that have not been absorbed may induce intestinal dysbiosis. An additional hypothesis is that bile acids exert a direct irritant effect on the colon wall.

It is noteworthy that there are numerous pathophysiological links between microscopic colitis (MC) and bile acid malabsorption (BAM). These conditions may share a similar molecular basis involving the Farnesoid X Receptor (FXR), Takeda G-Protein-Coupled Receptor 5 (TGR5), gut dysbiosis, and the Apical Sodium-Dependent Bile Acid Transporter (ASBT) [59].

The Farnesoid X Receptor (FXR) is a key nuclear receptor for bile acids, primarily expressed in the proximal colon as well as the liver. This receptor regulates bile acid synthesis. In colonic cells, FXR activation leads to the induction of fibroblast growth factor 19 (FGF19), which inhibits CYP7A1, the enzyme responsible for bile acid synthesis. Additionally, FXR reduces the absorption of bile acids in the intestine. These mechanisms collectively prevent the harmful effects of bile acids in the gut. Reduced levels of FXR consequently result in increased bile acid concentrations, which may lead to bile acid-related diarrhoea, epithelial damage induced by bile acids, and subsequently, the development of microscopic colitis (MC) [60,61].

The Apical Sodium-Dependent Bile Acid Transporter (ASBT) is a crucial transporter in the enterohepatic circulation of bile acids. This protein mediates the absorption of bile acids into the portal circulation in the distal ileum. Inflammatory conditions associated with microscopic colitis (MC) can lead to a reduction in ASBT levels in the ileum, resulting in an increased concentration of bile acids in the colon and subsequent induction of diarrhoea. A similar situation occurs in type 1 BAM. These observations confirm the importance of differentiating between these conditions [59].

The Takeda G-Protein-Coupled Receptor 5 (TGR5) is a bile acid receptor coupled with a membrane G-protein. This receptor is activated by bile acids and is responsible for enhancing insulin sensitivity, reducing liver steatosis, regulating gallbladder filling, maintaining intestinal barrier integrity, and reducing inflammation by inhibiting NF-κB, which decreases the production of pro-inflammatory cytokines. Therefore, dysregulation of TGR5 by bile acids prevents the activation of the NF-κB pathway, resulting in an increased amount of pro-inflammatory cytokines in the colon wall, which may contribute to the development of conditions such as microscopic colitis (MC) [62,63].

MC and BAM may co-occur, which is associated with their aforementioned overlapping pathophysiological mechanisms. This possibility should be considered, particularly in cases of drug resistance. It is crucial to rule out MC in patients with BAM-related diarrhoea that do not respond to bile acid sequestrants, and conversely, to exclude BAM in patients with MC who do not respond to budesonide. Additionally, it is important to recognize that changes induced by MC may contribute to bile acid diarrhoea [59].

### 3.11. Appendicitis

The appendix exerts an immunomodulatory effect due to its status as a cluster of lymphoid tissue. It plays a role in the production of B lymphocytes and the storage of beneficial bacterial strains. In the course of appendicitis, the Th17 lymphocyte pathway, which is associated with MC, is activated (these lymphocytes infiltrate the lamina propria of the colon) [64].

A nationwide clinicopathological study ESPRESSO (Epidemiology Strengthened by histoPathology Reports in Sweden) was conducted in Sweden. The study included 14,520 cases of MC and was based on data from national registries linked to histopathology reports. This study showed that there was an increased risk of developing MC even 10 years after appendectomy. This risk was 50% higher compared to those who did not undergo this surgical procedure. In addition, the effect of appendicitis severity on the MC risk was determined. Patients who underwent surgery for uncomplicated appendicitis (i.e., without appendiceal rupture) had the highest risk of developing MC within the first year following the procedure. In contrast, those with complicated appendicitis (i.e., with appendiceal rupture) or who had incidental appendectomy were at the greatest risk of developing MC 5 to 10 years after the operation [64].

### 3.12. Socio-Economic Status

Socio-economic status was shown to play a role in MC occurrence. The results of the study conducted by Sonnenber et al. [65] showed that the prevalence of this disease decreases with increasing income and house value. In addition, a positive correlation was also observed between the prevalence of this disease and higher education [65].

### 3.13. Dietary Factors: Calcium as a Factor That Reduces Risk of MC

A study conducted by Sandler et al. [66] aimed to evaluate the effect of diet on the development of MC with a particular focus on the role of calcium intake. The researchers observed that higher dietary calcium intake was associated with a lower risk of developing MC. The study group that consumed the most calcium had a low risk factor of 0.22 for developing MC, while those consuming the least calcium had a risk factor of 1.0 for developing MC. The calcium dose that most significantly reduced the risk of MC was 1055.03 mg for males and 800.27 mg for females. Higher calcium intake may be related to the fact that calcium increases the abundance of *Actinobacteria*, which are responsible for maintaining the integrity of the intestinal barrier. In addition, calcium stimulates the synthesis of short-chain fatty acids (SCFAs), which are beneficial for colonocyte metabolism and may promote anti-inflammatory effects and protect against the development of MC.

## 4. Symptoms

MC is characterized by alternating remissions and exacerbations with chronic watery diarrhoea without blood, which occurs suddenly, persists until diagnosis, and resolves with the implementation of appropriate treatment [67,68]. This manifestation is frequently accompanied by urgency and faecal incontinence [1,67]. The typical patient passes between six and nine stools per day; however, the number of stools passed may exceed 10 per day [1]. Moreover, 27% of patients also experience frequent bowel movements at night [69]. Additional symptoms may include abdominal discomfort, occurring in up to 41% of patients, abdominal pain in 31–42%, weight loss in 31–42% of cases, as well as fatigue, nausea, and vomiting. The symptoms of MC are outlined in Table 1 [68,69].

While chronic diarrhoea is the hallmark symptom of MC, there have been instances where chronic constipation has been the primary clinical presentation. Constipation was observed in 18% of LC patients and in up to 39% of CC patients. Furthermore, some patients exhibited constipation following episodes of diarrhoea [6].

In more severe forms of microscopic colitis, electrolyte imbalance and dehydration may occur, necessitating subsequent hospitalization. The disease is characterised by frequent recurrences, occurring in 30% to 60% of patients [70].

Due to its nonspecific symptoms, which are also present in more common gastrointestinal disorders, like IBS, MC is rarely considered in the first place and is therefore often underdiagnosed. It is important to note that in cases where treatment for chronic, watery diarrhoea attributed to other causes proves ineffective, MC should be considered.

## 5. Diagnostic Methods

A diagnosis of MC is typically made based on a comprehensive medical history, including chronic, bloodless diarrhoea. This is then corroborated with colonoscopy and histopathological examination of the biopsy specimen. It is recommended that a colonoscopy be performed on all patients presenting with diarrhoea with a duration of more than four weeks [71].

In accordance with the United European Gastroenterology (UEG) and European Microscopic Colitis Group (EMCG) guidelines for MC, a complete ileocolonoscopy should be performed, with numerous biopsies taken for histopathological examination [7].

The endoscopic image is typically unremarkable, with only 38.8% of cases exhibiting macroscopic alterations such as mucosal redness, oedema, vascular changes, cat scratches, mucosal ulceration, and a “furrowed” or “mosaic” pattern [67,72].

There is a paucity of consensus regarding the optimal number and distribution of biopsy specimens obtained during colonoscopy. This is illustrated in the following table (Table 2).

The histopathological criteria for the diagnosis of MC (Table 3) remain a topic of debate, despite recent efforts to consolidate knowledge on the subject and reduce inconsistencies in diagnosis between different laboratories. In particular, Danish pathologists have made significant contributions to this field, including the introduction of the concept of incomplete microscopic colitis [75].

As alternative and less invasive diagnostic methods were sought, research was conducted to identify markers for the diagnosis of MC. To date, studies have been conducted on a number of potential biomarkers, including faecal calprotectin (FCP), faecal lactoferrin (FL), myeloperoxidase (MPO), eosinophil cationic protein (ECP), eosinophil peroxidase (EPX), and alpha1-antitrypsin. It is unfortunate that, thus far, none of the molecules have demonstrated sufficient accuracy in differentiating from other IBD and IBS to the extent that they can be used in clinical practice. Furthermore, none of the non-invasive methods currently available are capable of screening and monitoring patients with MC [76].

The differential diagnosis of microscopic colitis encompasses a range of conditions, including irritable bowel syndrome (IBS), amyloidosis, endocrine neoplasia, bile acid circulation disorders, laxative abuse, coeliac disease, and amyloidosis [4].

It is important to note that the symptoms of MC may be confused with IBS, as nearly 39.1% of patients with MC report IBS-like symptoms, which include, among others, abdominal pain associated with defecation and/or changes in stool consistency or frequent bowel movements [77].

In the context of differentiating the causes of diarrhoea, BAM should be considered. As noted by Costa et al. [78], although BAM may affect 25–30% of patients with chronic diarrhoea, it is also a frequently overlooked diagnosis, which can lead to excessive health burdens. The article by Berti et al. [79] discusses in detail the dilemma of whether empirical treatment using bile acid sequestrants (BASs) is sufficient, or whether diagnostic tests such as the measurement of C4 (7α-hydroxy-4-cholesten-3-one), FGF-19, or the SeHCAT test, which are currently regarded as the gold standard, should be performed before the treatment initiation. It was concluded that incorporating these tests could help better differentiate BAD (Bile Acid Diarrhoea) from other causes of diarrhoea, including MC, and improve treatment efficacy by avoiding the risk of false-positive and false-negative results in empirical treatment.

## 6. Complications

Despite the generally benign course of MC, studies have demonstrated an increased risk of mortality among patients diagnosed with CC or LC compared to healthy controls. This risk was observed to be 1.8%, 3.4%, and 5.1% at 5, 10, and 15 years, respectively. The increased mortality can be attributed to a number of factors, among which gastrointestinal infections and disorders are of particular significance (see Section 6.2 “Gastrointestinal Tract Complications”) [80].

### 6.1. Arteriosclerosis and Cardiovascular System Complications

The available evidence indicates that patients with MC are at an elevated risk of developing cardiovascular events when compared to the general population. In order to assess the risk of MC-related complications, the MACE (major adverse cardiovascular event) index was employed, which encompasses ischaemic heart disease, heart failure, stroke, and cardiovascular mortality. With the exception of the risk of mortality, the incidence of these diseases was found to be as much as 27% higher in patients with MC compared to controls. Microscopic colitis has been demonstrated to induce the production of pro-inflammatory cytokines, which consequently result in systemic inflammation, which is a major predictor of coronary heart disease and plays a significant role in the development of the atherosclerotic process, increasing the risk of cardiovascular events. Furthermore, patients with CC are at an elevated risk, with 33% of them experiencing cardiovascular complications, compared to 24% of those with LC. The reasons for the disparate frequency of complications between MC types remain unclear [81].

Forss et al. [81] demonstrated that metabolic disorders, including diabetes (18.1%), hypertension (13.8%), and dyslipidaemia (3.0%), were more prevalent in MC patients than in healthy controls, potentially due to chronic inflammation.

To assess the burden of atherosclerosis in patients with microscopic colitis (MC), Hong et al. [82] conducted a study evaluating the prevalence of atherosclerotic diseases, including coronary artery disease (CAD), peripheral artery disease (PAD), and cerebrovascular disease (CVD), among patients with MC. The study did not show an increased prevalence of CAD, PAD, or CVD in the evaluated group. However, risk factors such as smoking, hypertension, and hyperlipidaemia may increase the risk of atherosclerosis in MC patients. Further research is needed to accurately evaluate the risk of developing atherosclerosis in patients with MC, as the study by Hong et al. was the first to address this issue.

It is also worth mentioning that the overall risk of venous thromboembolism in patients diagnosed with IBD was nearly twice as high compared to the general population [83]. Furthermore, patients with IBD have been found to have a significantly increased risk of mesenteric ischemia, primarily due to thromboembolic occlusion of the superior mesenteric artery [55,83]. A case has been reported of a female patient with IBD who developed mesenteric vein thrombosis, leading to ischemia of the colonic wall [84]. Since chronic inflammation is a common feature of both IBD and MC, it can be suspected that both diseases may share a similar pathogenic mechanism in the development of atherosclerosis of the mesenteric arteries. However, there is a lack of scientific studies assessing whether a correlation exists between mesenteric artery atherosclerosis and the occurrence of MC.

### 6.2. Gastrointestinal Tract Complications

There is an elevated risk of mortality from gastrointestinal causes and gastrointestinal tract infections in the MC group relative to the control group. The mortality rate from gastrointestinal infections was found to be 1.4 times higher [80]. Additionally, there is a 1.6-fold increased risk of acute pancreatitis and a 17-fold higher susceptibility to developing IBD of later onset, although the underlying mechanism remains unclear [85,86].

### 6.3. Neoplasia and Malignancy

In comparison to other inflammatory diseases, microscopic colitis does not appear to be a predisposing factor for the development of gastrointestinal adenomas or cancers, including those of the lung, breast, and thyroid [87,88,89]. A study by Levy demonstrated that among patients with MC, the prevalence of tubular adenoma was 31%, serrated adenoma was 8%, and villous adenoma was 1%. These rates were comparable to the incidence of adenomas in healthy patients with diarrhoea other than the MC control group (31.4%, 7.2%, and 1%, respectively), indicating that MC does not increase the risk of colorectal adenomas [87].

Moreover, the majority of studies indicate that there is no elevated risk of colorectal cancer among patients diagnosed with MC [90,91]. On the other hand, Bergman et al. observed an 8% increase in cancer risk in the initial year of follow-up in a Swedish cohort study, but the risk did not increase with disease duration [92].

## 7. Treatment

The treatment of MC encompasses lifestyle modifications, including cessation of smoking, reduction in alcohol consumption, and elimination of risk factors. In accordance with the European guidelines for the treatment of MC, all medications with the potential to cause diarrhoea should be discontinued [7].

**Figure 1:**
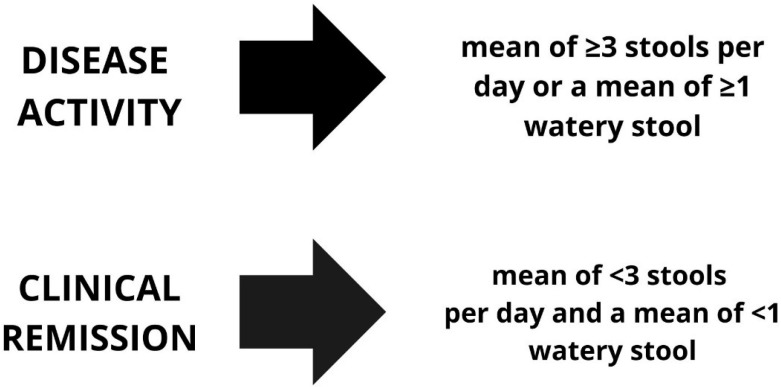
The criteria for diarrhoea remission and activity according to Hjörtswang [93].

The objective of treatment is to achieve remission. In order to ascertain whether a patient is in remission from MC, the Hjörtswang criteria (Figure 1) [93] are employed.

### 7.1. Budesonide

Budesonide represents the gold standard of treatment for all MC subtypes, as evidenced by the recommendations set forth by both the UEG and EMCG guidelines and the ASGE guidelines. It is recommended that 9 mg of budesonide be administered daily for a period of approximately 6–8 weeks to achieve clinical remission [7,94].

A review of the literature reveals that, following the aforementioned period, remission can be achieved in 81% of CC patients and in approximately 79–88% of LC patients [95,96,97].

Once remission has been achieved, maintenance therapy should be initiated. The recommended treatment regimen is 6 mg of budesonide per day for a period of six months. However, studies have demonstrated that an alternative regimen of 3 mg or 6 mg of budesonide per day for a period of 12 months has similar efficacy. It is important to note, however, that some patients may require doses higher than 6 mg daily to maintain remission, as demonstrated in the study by Fernandez-Bañares et al. [98].

Oral budesonide has a significant first-pass hepatic metabolism, which minimizes its systemic availability, with sustained immunosuppressive effects on the gastrointestinal tract similar to systemic corticosteroids. Therefore, it is considered a relatively safe and selective drug with primarily topical effects [99]. Furthermore, the use of the lowest effective dose enhances the safety profile and mitigates the likelihood of adverse effects associated with budesonide administration. The rare adverse effects linked with low-dose budesonide administration include osteoporosis, cataracts, steroid-induced diabetes mellitus, Cushing’s syndrome, an elevated risk of infection, mood disorders, hypertension, impaired wound healing capacity, and gastric and duodenal ulcers [100].

The effectiveness of budesonide enemas in the treatment of chronic collagenous colitis (CC) was also investigated. The enemas were given at a dose of 2 mg/100 mL twice daily for two weeks, and then once a day for four weeks. After this treatment, histopathologic remission was observed in 81.8% of patients, along with a reduction in collagen layer thickness, a decrease in bowel movements to fewer than four per day, and improvement in stool consistency. It is important to acknowledge a potential limitation of the described study: it was conducted on a relatively small population. This observation highlights the necessity for further studies to be conducted on the use of budesonide enemas [101].

### 7.2. Bile Acid Sequestrants (BASs): Cholestyramine, Colesevelam, and Colestypol

The efficacy of bile acid sequestrants (BASs) in achieving remission has been demonstrated in several studies. The studies revealed that approximately 66–76% of cases achieved remission following BAS treatment [102,103]. Furthermore, the results demonstrated that BASs can be used in order to reduce the dosage of corticosteroids in patients who require high doses of the latter to maintain remission. Tome et al. [102] also proposed that BASs could be considered as a second-line therapy for patients who are unable to tolerate or unresponsive to budesonide therapy.

In accordance with the UEG and EMCG guidelines [7], the administration of bile acid-binding drugs is advised for patients with MC who present with bile acid-related diarrhoea, as determined by SeHCAT studies. Conversely, the ASGE guidelines [94] do not consider BAS use in MC. 

### 7.3. Biological Drugs

The potential use of anti-TNF-α (anti-tumour necrosis factor) biologic drugs in the treatment of MC is being explored given that the inhibition of TNF-α has been demonstrated to reduce the expression of acute phase proteins and associated inflammation [104,105,106].

It has been demonstrated that following a 12-week course of treatment with infliximab (IFX), between 63 and 71.4% of patients achieved remission, while between 25 and 85.7% demonstrated a clinical response. In contrast, the data indicate that after adalimumab (ADA), remission was achieved in 40–42.9% of patients and a further 0–40% demonstrated a clinical response. It has been proposed that anti-TNF-α therapy may be an effective treatment option for patients who have demonstrated resistance to budesonide [107,108]. In a study by Daferera et al. [107], long-term remission was achieved in 16% of cases with the use of biologics. Additionally, it was observed that MC patients who were maintaining remission with anti-TNF-α therapy often required higher doses or a shortened interval between the subsequent doses.

Vedolizumab (VDZ) is an inhibitor of integrin α4β7, which blocks its interaction with MAdCAM-1. This results in the inhibition of T cell colonisation of the gut, thereby reducing symptoms and histological changes in MC. Furthermore, the interaction of α4β7 with MAdCAM-1 is largely confined to the gut, thereby rendering the effect of VDZ gut-specific [109].

It has been shown that after VDZ treatment, clinical response was achieved in 73% of patients and clinical remission in 56% of patients [72]. It has been postulated that vedolizumab may prove more efficacious than TNF-α inhibitors and that it has a more favourable safety profile in the elderly, due to its selective effect on the gut. Furthermore, this selective effect has not been found to increase the risk of infection or tumourigenesis [110,111].

### 7.4. Thiopurines and Methotrexate (MTX)

Thiopurines (azathioprine and mercaptopurine) represent the most extensively researched pharmacological agents for the management of MC in patients exhibiting an inadequate response to budesonide. A retrospective study by Cotter et al. [112] demonstrated that 43% of patients achieved a complete response and 22% achieved a partial remission. In the event of failure of budesonide treatment, the UEG and EMCG guidelines recommend the use of thiopurines [7].

Despite demonstrating efficacy in inducing complete or partial responses in 75–84% of cases, as evidenced by certain studies [112,113], methotrexate (MTX) is not endorsed by the UEG and EMCG guidelines [7]. This is due to the limited number of studies conducted, which have included relatively small sample sizes. Additionally, in some studies, the efficacy of the treatment was found to be minimal [114].

### 7.5. Combined Therapy of Microscopic Colitis

The use of combination therapy during MC treatment is noteworthy. In patients on budesonide treatment, we may additionally use symptomatic treatment such as antidiarrhoeal drugs, e.g., loperamide or bismuth subsalicylate (however, it should be noted that its long-term use may result in neurotoxicity). On the other hand, if MC is accompanied by diarrhoea related to bile acid malabsorption, bile acid sequestrants may be considered, which are also useful for reducing budesonide use. Furthermore, when the disease is refractory to treatment, thiopurine may also be considered as the second-line treatment [2,72,103].

In the GETAID study, combination therapy with biological agents was administered to five patients: ADA combined with budesonide, or IFX combined with azathioprine. This treatment approach was initiated due to the persistence of diarrhoea following monotherapy with budesonide or azathioprine, or due to symptom recurrence after discontinuation or dose reduction in corticosteroids. As a result of these combination therapies, patients receiving ADA achieved clinical response after 52 weeks, while those treated with IFX attained histological remission after approximately 12 weeks. These outcomes suggest the potential efficacy of combination therapies; however, further studies on larger patient cohorts are required to definitively confirm their effectiveness in the treatment of MC [108].

### 7.6. Surgical Treatment

In accordance with the UEG and EMCG guidelines, curative surgery should be employed as a last-resort treatment option in cases where pharmacological intervention has failed to elicit a response [7].

A permanent ileostomy is advised since the restoration of the physiological pathway has been shown to result in relapse [106].

Alternative approaches include an anastomosis of an intestinal reservoir to the rectum or a partial colectomy. However, there are relatively few documented cases of such procedures in the literature to date [115].

Since surgical treatment for microscopic colitis (MC) is rarely performed, there are no well-defined data on the timing of such interventions. However, as previously mentioned, surgery is considered a last-resort option and is only used when the symptoms are persistent and all of the pharmacotherapy methods fail.

### 7.7. Summary of Treatments

In summarising the treatment methods for microscopic colitis (MC), it is important to emphasize that managing this condition can be challenging for both patients and clinicians. Treatment may encounter difficulties, such as resistance to therapy. Additionally, monitoring the disease is complicated, requiring numerous colonoscopies with biopsies.

## 8. Summary

MC occurs with a frequency similar to that of other IBDs (with some studies indicating a higher prevalence). Therefore, gastroenterologists, internists, and primary care physicians should consider the possibility of this disease among patients presenting with chronic diarrhoea without blood, especially associated with night bowel movements, which points to the non-functionality of the disease. Furthermore, the absence of macroscopic changes at colonoscopy in a patient with chronic diarrhoea should prompt endoscopists to perform numerous biopsies. Failure to do so results in the necessity for an additional invasive examination.

The principles of biopsy collection and the histopathological criteria for the diagnosis of MC remain a matter of debate. Currently, expert groups are engaged in intensive efforts to reduce discrepancies in this field across different countries.

The gold standard for the treatment of MC is the oral administration of budesonide. Given the high first-pass effect through the liver, the risk of adverse effects associated with this treatment is minimal.

Although microscopic colitis is a chronic inflammatory condition, it does not directly result in an increased risk of mortality or an elevated risk of colorectal cancer compared to other inflammatory bowel diseases. The extended awareness of this disease among clinicians is necessary for early diagnosis and appropriate management.

MC is an inflammatory bowel disease that, despite its increasing recognition, remains underappreciated in clinical practice. This is largely due to the nonspecific nature of its symptoms, such as chronic diarrhoea and abdominal pain, which are often mistaken for more common chronic diarrhoea-associated disorders. Additionally, the absence of distinctive macroscopic changes in colonoscopy delays the diagnosis. Low disease awareness among clinicians and incomplete epidemiological data contribute to the insufficient consideration of MC in differential diagnosis. Therefore, there is a strong need to enhance the awareness of MC among the clinical staff and improve diagnostic and treatment strategies.

## Figures and Tables

**Table 1:** Symptom prevalence among patients with MC.

MC Symptoms	Symptom Prevalence
Chronic, watery diarrhoea without blood	84–100% [67,68]
Rectal tenesmus	55% [1,67]
Night-time bowel movements	27–35% [67,69]
Faecal incontinence	26.3% [66]
Weight loss	31–42% [68,69]
Abdominal pain	32–41% [68,69]
Nausea	3% [68]

**Table 2:** Recommendations for the number of biopsy specimens to be taken and the optimal location for biopsy collection.

Source	Number of Biopsy Specimen and Collection Site
UEG and EMCG 2021 guidelines [7]	A collection of multiple biopsies from the left and right colon.
ASGE 2013 guidelines [73]	A total of two biopsies should be collected from each colonic segment, including the ascending colon, transverse colon, descending colon, and sigmoid colon.
Malik et al. [74]	Three biopsies should be taken from the ascending colon and three from the descending colon.

ASGE: American Society of Gastroenterology; UEG and EMCG: United European Gastroenterology and European Microscopic Colitis Group.

**Table 3:** Histopathological criteria for MC according to the UEG and EMCG guidelines.

MC Features	CC	LC	MCi
Characteristic feature	Thickening of the collagen band > 10 μm, which may be irregularly thicker at the periphery (vertical positioning of the biopsy is recommended to better visualise the collagen band)	>20 IEL per 100 epithelial cells (IHC staining is recommended to facilitate counting)	CCi: thickening of subepithelial collagen band > 5 μm but less than 10 μm LCi: >10 IEL but less than 20 IEL per 100 epithelial cells and normal collagen band
Damage to the surface epithelium	Focal detachment from basement membrane, flattening, less mucus	Flattening, reduced mucin, and vacuolisation (less well expressed than in CC)	
Inflammatory plaque infiltration	Composed of plasma cells, lymphocytes, eosinophils, mast cells, and neutrophils	Predominance of lymphocytes and plasma cells; eosinophils and neutrophils sometimes present	
Paneth cell metaplasia	Rarely	Rarely	
Inflammation of the crypts	Rarely	Rarely	

CC—collagenous colitis, CCi—incomplete collagenous colitis, IEL—intraepithelial lymphocyte, IHC—immunohistochemistry, LC—lymphocytic colitis, LCi—incomplete lymphocytic colitis, and MCi—incomplete microscopic colitis.

## Data Availability

Not applicable.
